# Role of CCR2^+^ Myeloid Cells in Inflammation Responses Driven by Expression of a Surfactant Protein-C Mutant in the Alveolar Epithelium

**DOI:** 10.3389/fimmu.2021.665818

**Published:** 2021-04-22

**Authors:** Alessandro Venosa, Sophie Cowman, Jeremy Katzen, Yaniv Tomer, Brittnie S. Armstrong, Surafel Mulugeta, Michael F. Beers

**Affiliations:** ^1^ Department of Pharmacology and Toxicology, University of Utah College of Pharmacy, Salt Lake City, UT, United States; ^2^ Pulmonary, Allergy, and Critical Care Division, Department of Medicine, Perelman School of Medicine, University of Pennsylvania, Philadelphia, PA, United States; ^3^ PENN-CHOP Lung Biology Institute, University of Pennsylvania, Perelman School of Medicine, Philadelphia, PA, United States

**Keywords:** alveolar type-2 cell, Sftpc I73T surfactant protein-C I73T mutant, idiopathic pulmonary fibrosis, chemokine receptor-2, monocyte-derived alveolar macrophages, acute exacerbation of PF

## Abstract

Acute inflammatory exacerbations (AIE) represent precipitous deteriorations of a number of chronic lung conditions, including pulmonary fibrosis (PF), chronic obstructive pulmonary disease and asthma. AIEs are marked by diffuse and persistent polycellular alveolitis that profoundly accelerate lung function decline and mortality. In particular, excess monocyte mobilization during AIE and their persistence in the lung have been linked to poor disease outcome. The etiology of AIEs remains quite uncertain, but environmental exposure and genetic predisposition/mutations have been identified as two contributing factors. Guided by clinical evidence, we have developed a mutant model of pulmonary fibrosis leveraging the PF-linked missense isoleucine to threonine substitution at position 73 [I73T] in the alveolar type-2 cell-restricted Surfactant Protein-C [SP-C] gene [*SFTPC*]. With this toolbox at hand, the present work investigates the role of peripheral monocytes during the initiation and progression of AIE-PF. Genetic ablation of CCR2^+^ monocytes (SP-C^I73T^CCR2^KO^) resulted in improved lung histology, mouse survival, and reduced inflammation compared to SP-C^I73T^CCR2^WT^ cohorts. FACS analysis of CD11b^+^CD64^-^Ly6C^hi^ monocytes isolated 3 d and 14 d after SP-C^I73T^ induced injury reveals dynamic transcriptional changes associated with “Innate Immunity’ and ‘Extracellular Matrix Organization’ signaling. While immunohistochemical and *in situ* hybridization analysis revealed comparable levels of *tgfb1* mRNA expression localized primarily in parenchymal cells found nearby foci of injury we found reduced effector cell activation (C1q, iNOS, Arg1) in SP-C^I73T^CCR2^KO^ lungs as well as partial colocalization of *tgfb1* mRNA expression in Arg1^+^ cells. These results provide a detailed picture of the role of resident macrophages and recruited monocytes in the context of AIE-PF driven by alveolar epithelial dysfunction.

## Introduction

Pulmonary fibrosis (PF) is a devastating degenerating disease characterized by failure to properly resolve inflammation, heterogeneous disruption of alveolar and bronchiolar architecture, and irreversible scarring (1–3). Despite the intrinsic ability of the lung parenchyma to withstand repeated bouts of injury, persistent and widespread stress induced by endogenous (functional mutations) and/or exogenous (infection, toxicant exposure) sources promotes aberrant epithelial-immune and epithelial-mesenchymal communication. In this context, inflammation has been widely studied as an essential aspect of fibrogenic scarring and PF progression, with so called “acute inflammatory exacerbations” (AIE) strongly linked to lung function decline and mortality (4–6). Both clinical and experimental evidence indicate that mononuclear myeloid cell function reflects disease outcome, whereby disproportionate mobilization of peripheral monocytes, as well as their persistence in the lung as monocyte-derived alveolar macrophages, are associated with poor outcome (7–10).

Our understanding of the phenotype and function of resident and monocyte-derived macrophages and infiltrating monocytes in the context of chronic injury and fibrosis has been revolutionized in the past decade (8, 10–16). As a result, a growing research area is now dedicated to comprehend the interplay between ontogeny and polarization from birth to adulthood, in healthy and disease state (16–19). Leveraging chemical-induced fibrosis (i.e., bleomycin, asbestos) and innovative lineage tracing systems defined peripheral monocytes as a dynamic mixture of populations that has the capacity to differentiate into alveolar macrophage-like cells, yet remains incompetent in their ability to terminate/resolve inflammation, a function central to “true” resident alveolar macrophages (9, 14). With this in mind, it is essential to comprehend monocyte biology to fully appreciate their role in injury resolution and tissue remodeling occurring during AIE-PF (9, 20).

The etiological, temporal, and spatial complexity of PF has thus far represented an almost insurmountable roadblock to overcome. Epidemiological observation of familial cohorts of PF supports the notion that parenchymal mutations contribute to PF pathogenesis and progression, and provides a workable platform to develop translationally relevant models of PF (2, 21–23). In particular, mutations associated with key functional genes (i.e., telomere function, or pulmonary surfactants) are shown to robustly generate a fibrotic phenotype (3, 24). The most common mutation in the alveolar epithelial-restricted gene encoding for surfactant protein C (SP-C), the missense substitution g.1286T>C leading to isoleucine to threonine substitution at position 73 in the SFTPC proprotein (“SP-C^I73T^”), has been linked to extensive tissue remodeling driven by aberrant macroautophagy and mitophagy function (25, 26). We have previously demonstrated that allelic insertion of the mutant SP-C^I73T^ generates a viable strain producing hypomorphic levels of mutant SP-C (≈20% of SP-C^WT^ expression). This is associated with relatively moderate inflammation and lung remodeling (23). Thanks to its inducible nature, SP-C mutant levels can be tripled, thereby overwhelming epithelial cell capacity to cope with stress and systemic response. Previous evidence indicates that SP-C^I73T^ epithelial cells coordinate peripheral myeloid cell recruitment and activation, with their pro-inflammatory/pro-fibrotic activation is responsible for the propagation of the injury and fibrogenesis. In an effort to complement this dataset, we further provided initial proof of concept evidence that peripheral cell recruitment alters the trajectory of fibrotic disease induced by SP-C^I73T^ injury (27). Several clinical and experimental lines (SP-C^I73T^, bleomycin, radiation, asbestosis) of evidence draws strong correlation between excess monocyte mobilization and the outcome of inflammatory driven fibrosis, with specific chemokine axes responsible for the recruitment of distinct subsets (CCR2/CCL2, CCR4/CCL17, CX_3_CR1/CX_3_CL1) (28–32).

Building on these notions, the present work progressively narrows the cellular target responsible for lung remodeling during acute inflammatory exacerbations of PF from blood monocytes described in previous literature from our group and others (27, 33), to the Ly6C^+^ subset, and then the CCR2 subset. RNA-sequencing analysis of Ly6C^hi^ cells identified an early and persistent pro-inflammatory and pro-fibrotic phenotype during SP-C^I73T^ induced injury, while targeted depletion of the CCR2 subset (SP-C^I73T^CCR2^KO^) demonstrates that these cells are primarily involved in coordinating the degree of tissue damage downstream of the injury cue. Furthermore, use of *in situ* hybridization techniques provide evidence for spatial localization of activated immune cells with respect to fibrotic foci (*tgfb1*). Together, these data deliver an important piece of the puzzle, highlighting the contribution of specific monocyte/macrophages subsets in the initiation and progression of PF exacerbation.

## Materials and Methods

### Reagents

Tamoxifen (non-pharmaceutical grade) was purchased from Sigma-Aldrich (St Louis, MO). Giemsa cytological stain was purchased from Sigma-Aldrich. Antibodies used for *in situ* hybridization, immunohistochemical and flow cytometric analysis were: *tgfb1* (Cat # 407751, Advanced Cell Diagnostics, ACD); RFP (Cat # ab62341; 1:1000, Abcam, Cambridge, MA); Arg1 (Cat # ab91279; 1:1500, Abcam); iNOS (Cat # ab15323; 1:200, Abcam); CD64 (Cat # bs-3511R; 1:250, Bioss Antibodies, Woburn, MA); CCR2 (Cat # EPR20844; 1:400, Abcam); phospho-SMAD2/3 (Cat # PA5-37636; 1.500, Invitrogen CX_3_CR1 (Cat # bs-1728R; Bioss Antibodies); CD64 (Cat # bs-3511R; Bioss Antibodies); CD125/IL-5RA (Cat # bs-2601R, Bioss Antibodies); C1q (Cat # A0136; 1:500, Dako/Agilent Technologies, Santa Clara, CA); CD16/32 (clone 93; eBiosciences, San Diego, CA), CD11b (clone # M1/70; eFluo450, eBiosciences); Fixable Viability dye (Cat # 65-0865-14; eFluo780, eBiosciences); SigF (clone S17007L; PE-CF594, BD Biosciences, San Jose, CA); CD45 (clone 30-F11; PerCP5.5, Biolegend, San Diego, CA); CD11c (clone # N418; BV705, Biolegend); Ly6G (clone # 1A8; AF700, Biolegend); Ly6C (clone HK1.4, BV510, Biolegend); CD64 (clone X54-5/7.1; PE/Cy7, Biolegend); CD43 (clone # S11; PE, Biolegend); CD3 (clone # 17A2; BUV395, Biolegend). All other reagents were purchased from Thermo Fisher Scientific, Inc. (Waltham, MA), or Sigma-Aldrich.

### Murine Model of SP-C^I73T^ Induced Lung Injury

Tamoxifen inducible SP-C^I73T^ mice were generated as previously reported (9). Briefly, the SP-C^I73T^ founder line (expressing a Neomycin cassette) was crossed with a mouse line expressing an estrogen receptor (ER)-2 controlled Flp-O recombinase strain knocked into the Rosa26 locus (Jackson Laboratory, Bar Harbor, ME) to generate the inducible-SP-C^I73T^Flp line. adult homozygote SP-C^I73T^Flp mice received tamoxifen (175 mg/kg in corn oil, oral gavage) at 8-12 weeks of age. Both male and female animals were used for the studies. Control groups mice are represented as pooled data from tamoxifen treated SP-C^I73T^ not expressing Flp-O recombinase or oil (vehicle) treated Flp-O expressing SP-C^I73T^ mice. To knock out monocyte subsets, these mice were then crossed to homozygosity with CCR2^KO^ (Stock No: 004999, Jackson Laboratories) and CCR2^RFP^ (Stock No: 017586, Jackson Laboratories) lines. All mice were housed under pathogen free conditions in AALAC approved barrier facilities at the Perelman School of Medicine (University of Pennsylvania), and Skaggs College of Pharmacy, University of Utah. All experiments were approved by the Institutional Animal Care and Use Committee at the University of Utah and Pennsylvania.

### Lung Histology, Histochemistry, and *In Situ* Hybridization

Whole lungs were fixed by tracheal instillation of 10% neutral buffer formalin at a constant pressure (25 cm H_2_O). Following paraffin embedding, 6 µm sections were cut and stained with Hematoxylin & Eosin (H&E) by the Associated Regional and University Pathologists Inc., at the University of Utah. Immunostaining of deparaffinized tissue sections was performed as previously described (34). Briefly, after antigen retrieval using citrate buffer (10.2 mM sodium citrate, pH 6.0, for 20 minutes) and quenching of endogenous peroxidase with 3% hydrogen peroxide in methanol (30 minutes), non-specific binding was blocked with 10% goat or rabbit serum according to primary antibody origin. Appropriate serum/IgG controls each diluted in blocking buffer were applied for overnight incubation at 4°C in a humidified chamber. Following incubation with biotinylated secondary antisera (Vectastain Elite ABC kit, Vector Labs, Burlingame, CA) for 30 minutes (room temperature), staining was visualized using a Peroxidase Substrate Kit DAB (Vector Labs) and counterstained with Harris Modified Hematoxylin (Thermo Fisher Scientific, Inc.). In other studies, *in situ* hybridization was performed prior to immunohistochemical staining. Briefly, paraffin embedded sections were deparaffinized in xylene and 100% EtOH. This was followed by peroxidase quenching in H_2_O_2_ (10’, away from light), antigen retrieval (RNAscope^®^ Target Retrieval Reagent, ACD), and protease IV treatment (RNAscope^®^ Protease IV Reagent, ACD). Tgfb1 probe was then incubated for 2 h in hybridization oven (40°C), a step followed by a series of signal amplification steps and chromogenic development as indicated by manufacturer protocol. Slides were then washed and immunohistochemistry blocking step resumed as described above.

### Bronchoalveolar Lavage Fluid (BALF) Analysis

BALF was collected from mice using five sequential lavages of 1 ml sterile saline and processed for analysis as previously described (9). Briefly, cell pellets obtained by centrifuging BALF samples at 400 × *g* for 6 minutes were re-suspended in 1 ml of PBS, and total cell counts determined using a NucleoCounter (New Brunswick Scientific, Edison, NJ). Differential cell counts were determined manually from BALF cytospins stained with modified Giemsa for 20 minutes to identify macrophages, lymphocytes, eosinophils and neutrophils.

### Multiplex Cytokine Analysis

First-return aliquots of cell-free BALF were analyzed for CCL2 levels using a Luminex platform (Millipore Sigma, Burlington, MA) by the Human Immunology Core at Perelman School of Medicine.

### Flow Cytometry and Cell Sorting for Identification of Immune Populations

Following BALF collection, lungs were cleared of blood by cardiac perfusion with saline solution, removed from the chest cavity, minced, and transferred into a 50 ml conical tube and incubated (37°C, 30’) in DMEM + 5% FBS + 2 mg/ml Collagenase D (Cat #11088866001, Roche, Indianapolis, IN). Digested lungs were passed through 70-μm nylon mesh to obtain a single-cell suspension, counted and mixed with ACK Lysis Buffer (Thermo Fisher Scientific) to remove any remaining red blood cells. BALF and tissue cell pellet (1X10^6^ cells) were resuspended in 100µl staining buffer (PBS+0.1% sodium azide) and incubated with anti-mouse CD16/32 antibody (Fc block, eBiosciences, San Diego, CA) for 10 min at 4°C to block nonspecific binding. This was followed by 30-minute incubation with fluorescently-tagged antibodies or appropriate isotype controls (0.25–1.5 µg/10^6^ cells) for 30 minutes (4°C). Cells were then spun and resuspended in staining buffer for viability staining (30 minutes at 4°C). Cells were fixed in 2% paraformaldehyde and analyzed with an LSR Fortessa (BD Biosciences, San Jose, CA) or FACS ARIA (BD Biosciences) for cell sorting experiments. Inflammatory monocytes (SigF^+^CD11c^-^CD11b^+^Ly6C^+^) were identified following forward and side scatter selection of singlet CD45^+^ viable cells. To ensure cell sorting of a purified population of monocytes pregating/exclusion of resident alveolar macrophages (SigF^+^ CD11b^-^CD11c^+^), eosinophils (SigF^int^CD11b^+^CD11c^-^), neutrophils (Ly6G^+^) and lymphocytes (CD3^+^), based on gating strategy modified from our group and others (27, 35, 36). Gating strategy is shown in **Supplementary Figure 1**. All analysis was performed using FlowJo software (FlowJo, LLC, Ashland, Oregon).

### RNA Sequencing Preparation and Analysis

Total RNA was extracted from fresh frozen cell pellets using Qiagen RNeasy Plus Universal mini kit following manufacturer’s instructions (Qiagen, Hilden, Germany). Extracted RNA samples were quantified using Qubit 2.0 Fluorometer (Life Technologies, Carlsbad, CA, USA) and RNA integrity was checked using Agilent TapeStation 4200 (Agilent Technologies, Palo Alto, CA, USA). RNA sequencing libraries were prepared using the NEBNext Ultra RNA Library Prep Kit for Illumina following manufacturer’s instructions (NEB, Ipswich, MA, USA). Briefly, mRNAs were first enriched with Oligo(dT) beads. Enriched mRNAs were fragmented for 15 minutes at 94°C. First strand and second strand cDNAs were subsequently synthesized. cDNA fragments were end repaired and adenylated at 3' - ends, and universal adapters were ligated to cDNA fragments, followed by index addition and library enrichment by limited-cycle PCR. The sequencing libraries were validated on the Agilent TapeStation (Agilent Technologies, Palo Alto, CA, USA), and quantified by using Qubit 2.0 Fluorometer (Invitrogen, Carlsbad, CA) as well as by quantitative PCR (KAPA Biosystems, Wilmington, MA, USA). The sequencing libraries were pooled and clustered on 1 lane of a flowcell. After clustering, the flowcell was loaded on the Illumina HiSeq4000 instrument according to manufacturer’s instructions. The samples were sequenced using a 2x150bp Paired End (PE) configuration. Image analysis and base calling were conducted by the HiSeq Control Software (HCS). Raw sequence data (.bcl files) generated from Illumina HiSeq was converted into fastq files and de-multiplexed using Illumina’s bcl2fastq 2.17 software. One mismatch was allowed for index sequence identification. Analysis of RNA counts was performed using R (3.6.3) (37). Differential gene expression analysis was conducted using the hciR package (38). Fast Gene Set Enrichment Analysis (fgsea) was used for gene set enrichment analysis with the Reactome database (39). Data were deposited in NCBI’s Gene Expression Omnibus (40) and are accessible through GEO Series accession number GSE166300 (https://www.ncbi.nlm.nih.gov/geo/query/acc.cgi?acc=GSE166300).

### Statistics

All data are presented with dot-plots and group mean ± SEM unless otherwise indicated. Statistical analyses were performed with Prism GraphPad 9.0 (GraphPad Software, San Diego, CA). Student’s t-test were used for paired data; for analyses involving multiple groups, one-way or two-way analysis of variance (ANOVA) was performed with *post hoc* testing as indicated. Survival analyses were performed using Log Rank (Mantel-Cox) test. In all cases statistical significance was considered at p *≤* 0.05.

## Results

### SP-C^I73T^ Induced Injury Is Linked to Monocyte Activation

**Figure 1:**
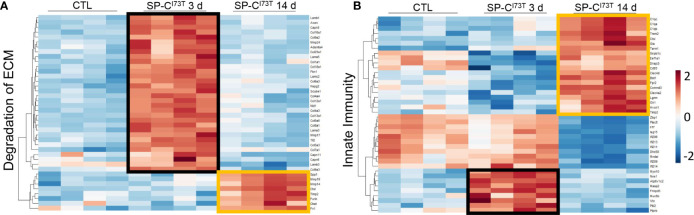
Extracellular matrix and innate immunity expression analysis of flow cytometry sorted CD11b^+^Ly6C^hi^ monocytes following SP-C^I73T^ induced injury. Differentially expressed genes (fold change > 1.5 and false discovery rate [q-value] < 0.05) were analyzed using Reactome database to highlight enriched signaling pathways. N = 4 was utilized in each study condition. **(A)** Heat-map showing expression of significantly regulated genes involved in ‘Degradation of Extracellular Matrix (ECM)’. **(B)** Heat-map showing expression of significantly regulated genes involved in ‘Innate Immunity’. Note black boxes represent genes uniquely expressed 3 d after SP-C^I73T^ injury; orange boxes represent genes uniquely expressed 14 d after SP-C^I73T^ injury.

**Figure 2:**
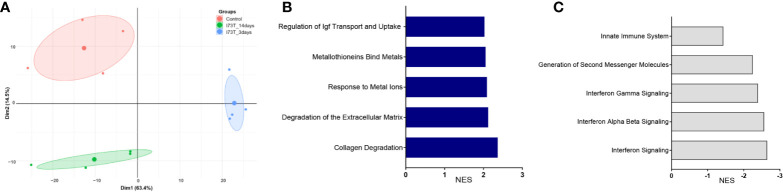
RNA sequencing analysis of flow cytometry sorted cells shows temporal dynamics in CD11b^+^Ly6C^hi^ monocytes function following SP-C^I73T^ induced injury. RNA-sequencing of cell sorted CD11b^+^Ly6C^hi^ monocytes from control (CTL, oil treated SP-C^I73T^ mice) or SP-C^I73T^ mice 3 d and 14 d after injury. Total RNA was prepared and analyzed by RNA-seq. N = 4 was utilized in each study condition. **(A)**, Two-dimension principle component analysis (PCA) of gene expression variance among monocytes accumulating in the lung in control (CTL) or SP-C^I73T^ mice 3 d and 14 d after injury. **(B)** Positively enriched pathways at 3 d post injury (normalized enrichment score, NES); **(C)** Negatively enriched pathways at 14 d post injury.

We have previously shown that lung injury generated by induction of mutant SP-C^I73T^ expression is accompanied by dynamic changes in SigF^+^CD11b^int^ resident alveolar macrophage and CD11b^+^Ly6C^hi^ infiltrating monocyte mobilization and activation (27). We therefore used comparable gating strategy to sort CD11b^+^Ly6C^hi^ inflammatory monocytes from collagenase digested tissue 3 d and 14 d post induction, times coordinated with initiation and peak of inflammatory exacerbations. Principle component analysis (PCA) revealed transcriptional variance (Dim1: 63,4%; Dim2: 14,5%) between the control group, consisting of oil treated SP-C^I73T^ mice, an acute inflammatory state (3 d post tamoxifen induced SP-C mutant induction) and late inflammation/early remodeling (14 d) (**Figure 1A**). Pathway analysis of CD11b^+^Ly6C^hi^ monocyte isolated 3 d post injury indicated transcriptional changes (positive and negative normalized enrichment score, NES) related to survival and extracellular matrix homeostasis (**Supplementary Figure 2**). By 14 d, signaling pathways associated with extracellular matrix remodeling displayed sustained positive NES scores (**Figure 1B**), while those associated with inflammatory activation (i.e., innate immunity, interferon signaling) showed a negative NES (**Figure 1C**). Further examination of significantly altered genes belonging to ‘Degradation of extracellular matrix’ gene set revealed metalloproteinases (*mmp17, mmp24*) and collagen genes (*col6a1, col1a1, col8a1, col4a3, col2a1*) to be significantly increased at 3 d (**Figure 2A**). By comparison, analysis of Ly6C^hi^ monocyte transcripts at 14 d highlighted downregulation of all the above-mentioned transcripts, paired with increases in a distinct subset of matrix/matrix degradation, including *spp1, mmp19, mmp14, timp2*, and *fn1* (**Figure 2A**, black boxes). Analysis of gene sets linked to ‘Innate immunity’ outlined expression signatures exclusive to 3 d (*nos1, frmpd3; masp2*) and 14 d (*c1qa/c, cd93, clec4a2;* and downregulated expression of *plac8, irf7, zbp1*) post injury (**Figure 2B**, black and orange boxes). Similar examination of ‘Interferon signaling’ *also identified genes distinctively expressed acutely (trim2/6/17, irf6) or at 14 d (socs3, and marked reductions in stat1, trim34a, irf7)* (**Supplementary Figure 3**, black boxes).

**Figure 3:**
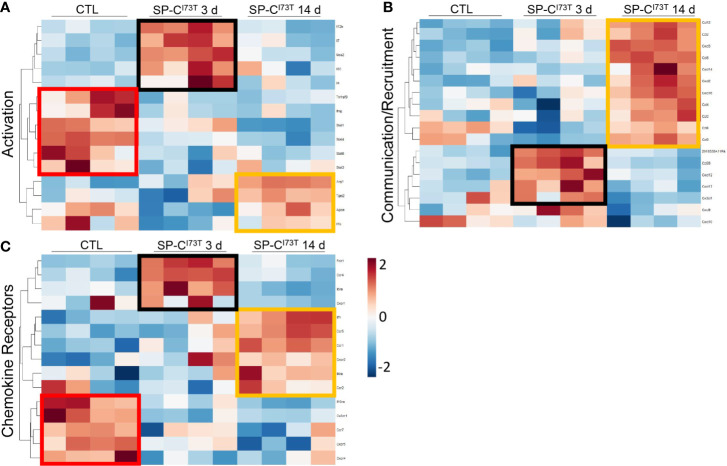
Targeted gene expression analysis of flow cytometry sorted CD11b^+^Ly6C^hi^ monocytes following SP-C^I73T^ induced injury. **(A-C)** Differential gene expression of factors associated with **(A)** activation, **(B)** cell communication/recruitment, or **(C)** chemokine receptor. Heat maps show significant genes. Gene selection was based on previous literature (9, 42, 43). Criteria for significance were set at fold change > 1.5 and false discovery rate (q-value) < 0.05. N = 4 was utilized in each study condition. Note red boxes represent genes uniquely expressed in control populations; black boxes represent genes uniquely expressed 3 d after SP-C^I73T^ injury; orange boxes represent genes uniquely expressed 14 d after SP-C^I73T^ injury.

As a complement to our unbiased pathway analysis, a gene list extrapolated from literature in pulmonary fibrosis examined dynamic changes in monocyte activation, recruitment, and surface receptor expression after SP-C^I73T^ induced injury (8, 9, 13, 14, 41) (**Figures 3A–C**). We found that Ly6C^hi^ monocytes isolated from control lung digests expressed high levels of transcription factors involved in the inflammatory response (*Stat1, Stat4* and *Stat5)*, displayed a unique chemokine receptor repertoire (*cx3cr1, ccr2, ccr7, cxcr4, cxcr5, il10ra*), and negligible levels of chemokine/cytokine ligands compared to cells isolated after SP-C^I73T^ induced injury (**Figures 3A–C**, red boxes). By comparison, Ly6C^hi^ monocytes accumulating in the lung 3 d post injury expressed *nos2, il12b* and *il7* (**Figure 3A**, black boxes) and a battery of chemokine/cytokine ligands *(ccl28, cxcl12, cxcl17, cx3cl1)* (**Figure 3B**, black boxes), and receptors (*folr1, ccr4* and *il5ra*) (**Figure 3C**, black boxes). This was further changed by 14 d, which was coordinated with high levels of anti-inflammatory genes (*arg1, tgm2, apoe*) recruitment factors (*ccl2/3/4/6/8/9/12, cxcl2/14/16*), as well as a unique receptor repertoire characterized by *il7r, ccr1, ccr5, il4ra* (**Figures 3A–C**, orange boxes).

### CCR2 Monocyte Depletion Reduced Injury and Inflammatory Induced by SP-C^I73T^ Expression

**Figure 4:**
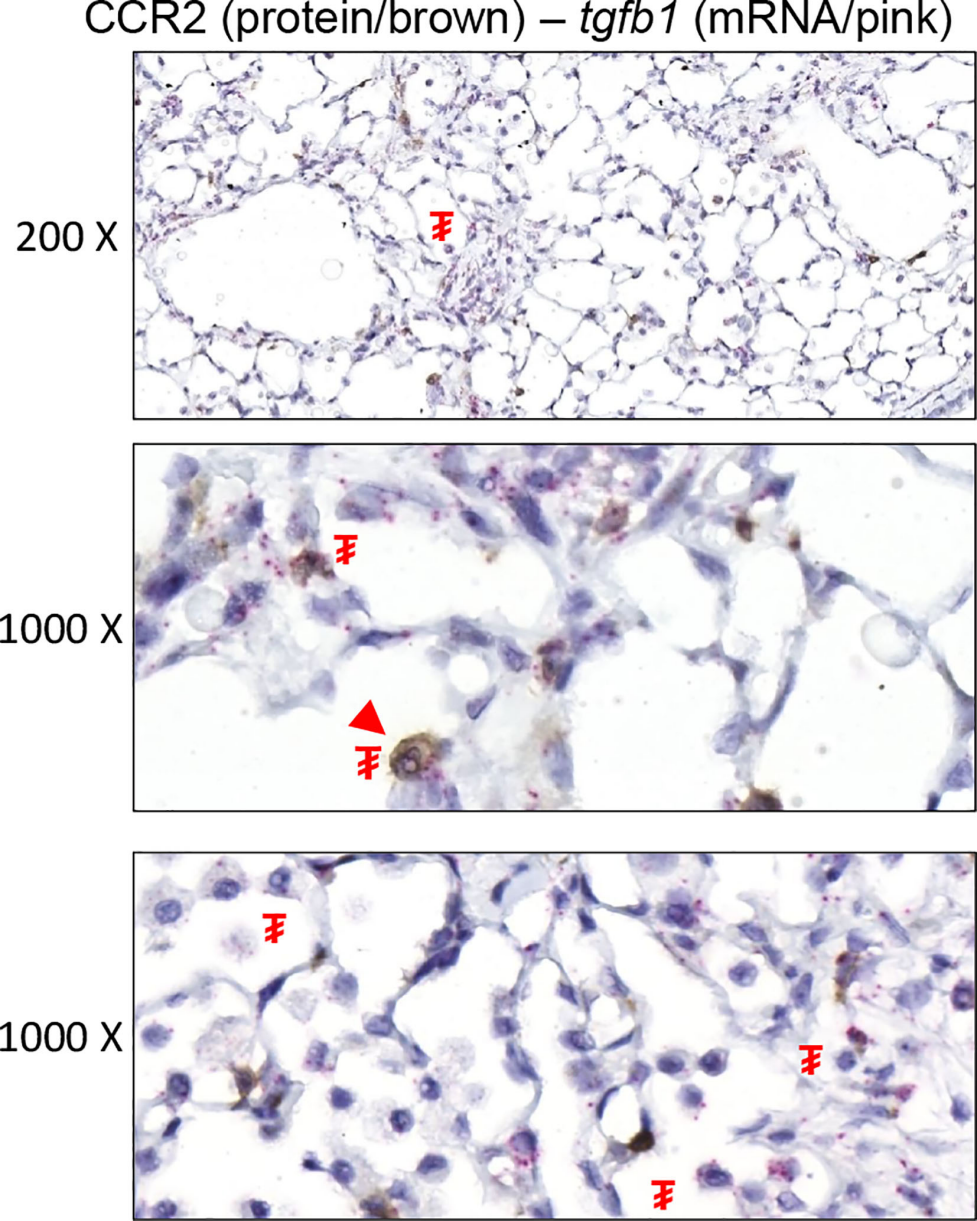
Tgfb1 expression in CCR2^+^ cells following SP-C^I73T^ induced injury. In situ hybridization (*tgfb1/pink*) combined with immunohistochemistry (CCR2/brown) analysis of SP-C^I73T^CCR2^WT^ lung 14 d after injury. ₮ indicates area expressing tgfb1; arrowhead indicates CCR2 staining cells. Representative 200x (top) and 1000x (bottom) images from N = 3 separate animals are shown.

**Figure 5:**
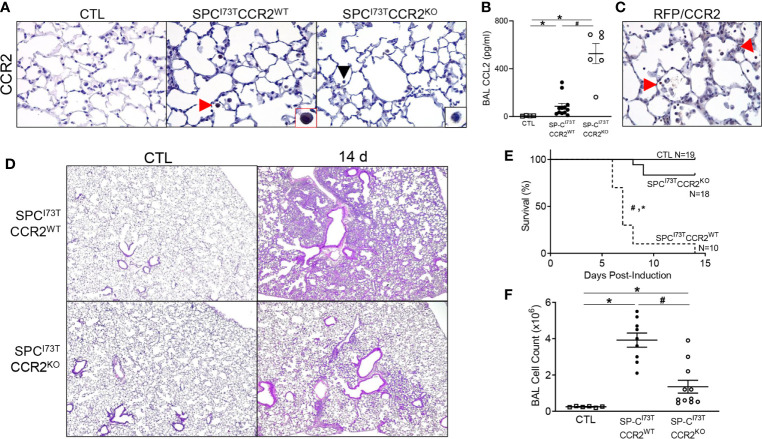
Effects of genetic CCR2 monocyte ablation on lung injury, survival and inflammation following SP-C^I73T^ mutant induced injury. **(A)** Immunohistochemical analysis of control (CTL, tamoxifen treated SP-C^WT^ or oil treated SP-C^I73T^ mice), SP-C^I73T^CCR2^WT^ and SP-C^I73T^CCR2^KO^ lung sections were immunostained with antibody to CCR2. Red arrowheads indicate myeloid cells staining for CCR2. Insets show magnified cells; box color matches that of respective arrowhead. Black arrowheads indicate negative CCR2 staining in mononuclear cells in SP-C^I73T^CCR2^KO^ cohorts. Images shown are representative of 3-5 animals per group. Magnification: 400x. **(B)** CCL2 ELISA of BAL fluid from control (CTL, tamoxifen treated SP-C^WT^ or oil treated SP-C^I73T^ mice), SP-C^I73T^CCR2^WT^ and SP-C^I73T^CCR2^KO^ mice 14 d following tamoxifen administration. Data are represented as mean ± SEM (N=4-13). **(C)** Histochemical analysis of SP-C^I73T^CCR2^RFP^ lung sections 14 d post injury immunostained with antibody to RFP/CCR2. Arrowheads indicate cells expressing the receptor **(D)** Hematoxylin & Eosin stained sections of control (CTL, tamoxifen treated SP-C^WT^, CCR2^KO^, or oil treated SP-C^I73T^ mice), SP-C^I73T^CCR2^WT^ and SP-C^I73T^CCR2^KO^ lungs 14 d post injury. Magnification: 400x. **(E)** Kaplan–Meier survival analysis from control (tamoxifen treated SP-C^WT^ or oil treated SP-C^I73T^ mice) SP-C^I73T^CCR2^WT^ and SP-C^I73T^CCR2^KO^. Mice found dead or displaying body weight loss equating >25% of stating weigh for 2 consecutive days. *p<0.05 compared to control mice; ^#^p<0.05 compared to SP-C^I73T^CCR2^WT^ mice by Log-Sum (Mantel-Cox) Rank test. **(F)** BAL fluid cell counts from control (CTL, tamoxifen treated SP-C^WT^ or oil treated SP-C^I73T^ mice), SP-C^I73T^CCR2^WT^ and SP-C^I73T^CCR2^KO^ (N =6-11) 14 d following SP-C^I73T^ induced lung injury. Data are represented as mean ± SEM. *p<0.05 compared to control mice; ^#^p<0.05 compared to SP-C^I73T^CCR2^WT^ mice by One-Way ANOVA, using Tukey post-hoc test.

Previous work conducted by our laboratory preliminarily linked early influx of peripheral myeloid cells to lung disease outcome following mutant SP-C induction (27). Rather than investigate the fibrogenic potential of the broader monocyte population, represented by Ly6C expression, we opted to limit our search to a subset of monocytes expressing CCR2, a marker linked with lung injury and fibrosis (33, 44). RNA *in situ* hybridization analysis indicated that CCR2^+^ cells do not express the master regulator of fibrosis, *tgfb1*; rather, we noted it to be predominant parenchymal (epithelial and mesenchymal), while CCR2^+^ cells accumulated in the proximity of these *tgfb1-*rich foci of injury (**Figure 4**). To test whether ablation of the CCR2^+^ pool results in disease modifying effects on SP-C^I73T^ induced injury, we opted to utilize a knock out model directly targeting CCR2 monocytes (SP-C^I73T^CCR2^KO^). We confirmed that CCR2^+^ cells accumulate in the lung following SP-C^I73T^ induced injury, and that this response is not observed in SP-C^I73T^CCR2^KO^ mice (black arrowhead, **Figure 5A**). Notably, despite CCR2 receptor depletion, levels of MCP1/CCL2 were significantly increased compared to CCR2^WT^ counterparts, whereas CCL17 and CX3CL1, chemokines also involved in monocyte recruitment, were not altered (**Figure 5B** and not shown). Histochemical analysis for RFP in lung sections isolated from mice expressing red fluorescent protein in lieu of CCR2 (SP-C^I73T^CCR2^RFP^) 14 d post injury also revealed accumulation of RFP/CCR2 positive cells, thus indicating that monocytes expressing CCR2 may be recruited through mechanisms independent of CCR2 (**Figure 5C**). Next, we performed histopathological analysis of SP-C^I73T^CCR2^WT^ and SP-C^I73T^CCR2^KO^ lungs 14 d post injury. Unsurprisingly, in control conditions (SP-C wild type and oil treated SP-C^I73T^ mutant lines) CCR2 monocyte ablation was not associated with any architectural alterations (**Figure 5D**, left panels). As previously described, tamoxifen induction of SP- C^I73T^CCR2^WT^ mice was associated with extensive perivascular and alveolar inflammatory cell infiltration and alveolar architecture disruption at 14 d **(** **Figure 5D**, right panels) (23, 27). By comparison, both inflammatory cell clustering within the alveolar space and early fibrotic remodeling were lessened in CCR2 depleted mice. Consistent with these observations, CCR2 monocyte ablation was also linked to reduction in animal mortality (100% SP-C^I73T^CCR2^WT^ vs. 16.7% SP-C^I73T^CCR2^KO^, at 2 weeks) and bronchoalveolar lavage (BAL) cell count (**Figures 5E, F**).

### CCR2 Monocyte Depletion Alters Inflammatory Cell Recruitment and Activation

**Figure 6:**
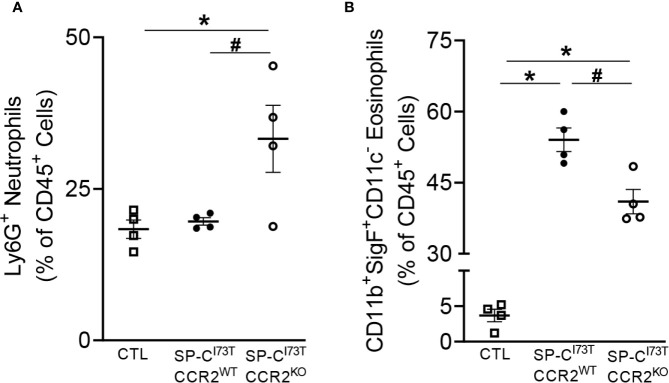
CCR2 monocyte depletion results in altered polymorphonucleated cell influx following SP-C^I73T^ induced injury. Changes in relative abundance of tissue **(A)** Ly6G^+^ neutrophils and **(B)** SigF^+^CD11c^-^CD11b^+^ eosinophils isolated by enzymatic digested (collagenase D) from control (CTL, tamoxifen treated SP-C^WT^ or oil treated SP-C^I73T^ mice), SP-C^I73T^CCR2^WT^ and SP-C^I73T^CCR2^KO^ 14 d following injury. Data are represented as mean ± SEM (N = 4). All analysis was considered significant *p<0.05 compared to control mice; ^#^p<0.05 compared to SP-C^I73T^CCR2^WT^ mice by One-Way ANOVA, using Tukey post- hoc test.

Flow cytometric analysis of lung tissue digest and manual cytospin counts of BAL cells revealed dynamic changes in neutrophil and eosinophil accumulation following CCR2 monocyte depletion. Flow cytometric analysis of tissue digests revealed no changes in the relative abundance of alveolar macrophages (SigF^hi^CD11b^lo^CD11c^+^) and lymphocytes (CD3^+^) in SP-C^I73T^CCR2^KO^ mice. Comparatively, accumulation of Ly6G^+^ neutrophils almost doubled in SP-C^I73T^CCR2^KO^ mice (18.35 ± 1.5% in CTL; 19.63 ± 0.61% in SP-C^I73T^CCR2^WT^; 33.25 ± 5.6% in SP-C^I73T^CCR2^KO^), while overall eosinophilia was reduced at 14 d post injury (**Figures 6A, B**).

**Figure 7:**
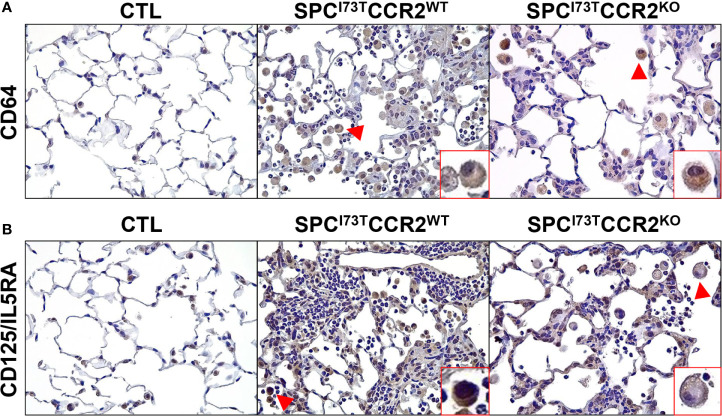
Effects of CCR2 monocyte ablation on monocyte/macrophage maturation and recruitment following SP-C^I73T^ induced injury. Histochemical analysis of control (CTL, tamoxifen treated SP-C^WT^ or oil treated SP-C^I73T^ mice), SP-C^I73T^CCR2^WT^ and SP-C^I73T^CCR2^KO^ lung sections 14 d post injury immunostained with antibody to **(A)** CD64 and **(B)** CD125/IL5RA. Binding was visualized using a Vectastain kit. Arrowheads indicate cells expressing the receptor. Insets show magnified cells; box color matches that of respective arrowhead. Original magnification, 400x; inset magnification, 750x. Representative sections from 3 mouse/group are shown.

**Figure 8:**
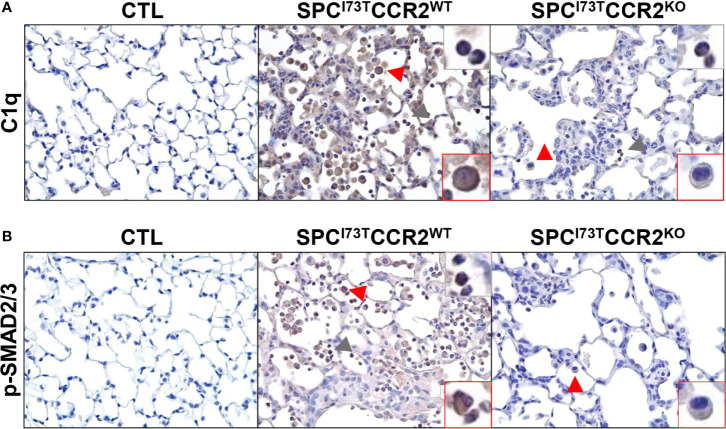
Effects of CCR2 monocyte ablation on inflammatory and fibrotic pathways following SP-C^I73T^ induced injury. Histochemical analysis of control (CTL, tamoxifen treated SP-C^WT^ or oil treated SP-C^I73T^ mice), SP-C^I73T^CCR2^WT^ and SP-C^I73T^CCR2^KO^ lung sections 14 d post injury immunostained with antibody to **(A)** C1q and **(B)** p-SMAD2/3. Binding was visualized using a Vectastain kit. Red arrowheads indicate macrophage expression; grey arrowheads indicate non-macrophage expression. Insets show magnified cells; box color matches that of arrowhead. Original magnification, 400x; inset magnification, 750x. Representative sections from 3 mice/group are shown.

Immunohistochemical analysis, alone or in combination with *in situ* hybridization, was used to examine changes in myeloid cell maturation (CD64, **Figure 7A**), surface receptor repertoire (IL5R and CX_3_CR1 **Figure 7B** and **Supplementary Figure 4**), and lung inflammatory state (iNOS, Arg1, *tgfb1*, **Figures 8** **,** **9**) following depletion of CCR2 monocytes in SP-C^I73T^ mice. Ablation of CCR2^+^ cells dampened the number of CD64^+^ mature macrophages accumulating within foci of injury (arrowheads, **Figure 7A** and **Supplementary Figure 5A**). Similarly, SP-C^I73T^CCR2^KO^ mice significantly reduced the abundance of macrophages expressing IL-5RA receptor (CD125), a response likely to be guided by increases in IL-5 expression and secretion by epithelial cells during SP-C^I73T^ induced injury (**Figure 7B** and **Supplementary Figure 5B**) (27). Differential expression of chemokine receptors allows us to define functionally distinct monocyte/macrophage populations. In this context, CCR2 and CX_3_CR1 have been used to discriminate the mobilization of Ly6C^hi^ monocytes from the bone marrow and their accumulation at the site of injury, respectively (45). Ablation of CCR2^+^ monocyte did not affect accumulation of CX_3_CR1^+^ cells at 3 d post injury; by 14 d, we noted increases in the number of CX_3_CR1 expressing cells but not the relative intensity in individual cell expression (**Supplementary Figure 4**, **5C**). There was no difference in monocytes/macrophages maturation and activation between CCR2 wild type and knock outs in non-remodeled regions of the lung (data not shown).

**Figure 9:**
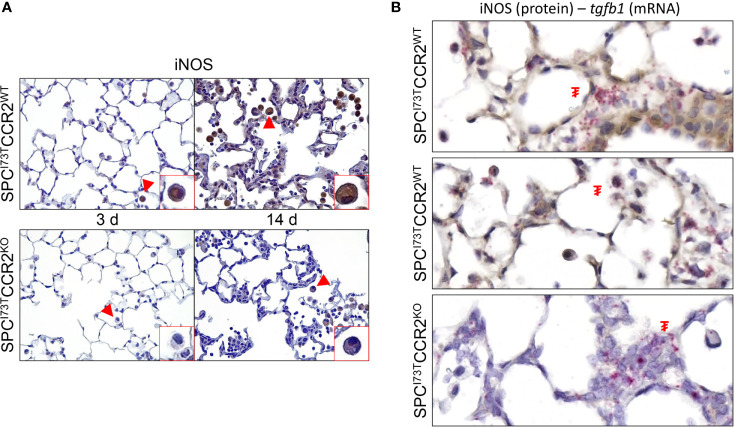
Effects of CCR2 monocyte ablation on pro-inflammatory activation following SP-C^I73T^ induced injury. Histochemical analysis alone or in combination with *in situ* hybridization of SP-C^I73T^CCR2^WT^ and SP-C^I73T^CCR2^KO^ lung following SP-C^I73T^ induced injury. Sections were immunostained with antibody to **(A)** iNOS. Binding was visualized using a Vectastain kit. Insets show magnified cells; box color matches that of arrowhead. Original magnification, 400x; inset magnification, 750x. **(B)** IHC + *in situ* hybridization (iNOS^+^ *Tgfb1)*. mRNA visualization is shown in pink. Protein expression was visualized using a DAB Vectastain kit (brown). Arrowheads indicate cells expressing the receptor. **₮** indicate mRNA expressing cells. Original magnification, 1000x. Representative region is shown (N = 3).

**Figure 10:**
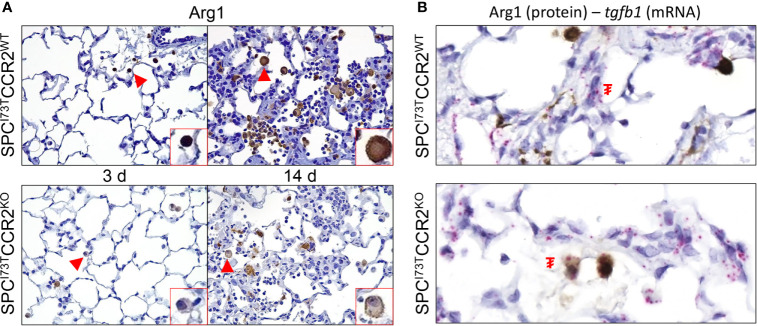
Effects of CCR2 monocyte ablation on anti-inflammatory activation following SP-C^I73T^ induced injury. Histochemical analysis alone or in combination with *in situ* hybridization of SP-C^I73T^CCR2^WT^ and SP-C^I73T^CCR2^KO^ lung following SP-C^I73T^ induced injury. Sections were immunostained with antibody to **(A)** Arg1 or **(B)** Arg1+*Tgfb1*. mRNA visualization is shown in pink. Protein expression was visualized using a DAB Vectastain kit (brown). Arrowheads indicate cells expressing the receptor. **₮** indicate mRNA expressing cells. Original magnification, 1000x. Representative region is shown (N = 4).

We then shifted our histochemical analysis towards macrophage activation. Guided by our RNA-sequencing results, we found increases in expression of the inflammation-linked complement component 1q (C1q) in all immune subsets and parenchymal cells (**Figure 8A**). By comparison, phosphorylation of SMAD2/3, a protein downstream of the TGFβ1 signaling pathway, was restricted to inflammatory cells (**Figure 8B**). Expression of these markers in CCR2 depleted cohorts revealed almost complete ablation of these responses, with sparse polymorphonucleated cell expression of C1q (**Figure 8A**, grey arrowhead). Consistent with these results, we analyzed expression of the canonical pro-inflammatory activation marker, iNOS. While its expression was negligible at baseline (**Supplementary Figure 6A**), iNOS expression was progressively amplified in SP-C^I73T^CCR2^WT^ lungs at 3 d and 14 d post injury (**Figure 9A**, top panels). Notably, we found no iNOS expression at 3 d in SP-C^I73T^CCR2^KO^ lungs; 14 d post injury, parenchymal and inflammatory cell expression was visible but significantly reduced (**Figure 9A**). Analysis of iNOS protein expression with *in situ* hybridization demonstrated accumulation of iNOS^+^ cells in proximity of foci of injury enriched in *tgfb1* mRNA expression (**Figure 9B**). As described in **Figure 9A**, no iNOS^+^ cells were visible in SP-C^I73T^CCR2^KO^, but *Tgfb1* expression was not affected by CCR2 monocyte ablation. We also found higher Arg1 expression (both number of expressing cells and relative intensity) in SP-C^I73T^CCR2^WT^ mice, both at 3 d and 14 d after SP-C^I73T^ induced lung injury (**Supplementary Figure 6B** and **Figure 10A**). Analysis of fibrotic foci, identified by higher intracellular *tgfb1* density, revealed no colocalization with ARG1positive cells in SP-C^I73T^CCR2^WT^ mice, but we observed a number of double positive cells in the CCR2 knock out cohorts (**Figure 10B**).

## Discussion

Pulmonary fibrosis represents the end-result of an aberrantly resolved inflammatory state. Early signals of stress are initially coordinated by the lung parenchyma, whereby both resident and peripheral immune subsets are central in the subsequent remodeling. These so-called “acute inflammatory exacerbations” are centrally responsible for propagating the injury and triggering rapid histological and functional decline through scarring and alveolar remodeling (honeycombing), events that ultimately accelerate patient death (4). Although we broadly comprehend these responses, there are substantial knowledge gaps related to the specific mechanisms by which exacerbations are initiated, the factors regulating individual thresholds of disease, and the nuances of studying a spatially heterogeneous injury. As a result of these obstacles, the first line of therapy against PF relies on broad-spectrum agents (corticosteroids, cytokine modulators, anti-fibrotics) that only target the symptoms (46–48). To refine our approach to disease interventions during disease defining processes such as exacerbations of PF, it is pivotal that we dynamically identify and functionally characterize inflammatory cell populations. In these studies, we provide transcriptional profiling of Ly6C^+^ peripheral myeloid populations during initiation and progression of inflammatory exacerbations triggered by excess mutant SP-C^I73T^. Furthermore, we provide evidence that a small subpopulation of bone marrow monocytes identified by their CCR2 expression, (CCR2^+^) monocytes represent a valuable candidate responsible for shifting disease trajectory.

The vast majority of experimental models of PF rely on exogenous stressors (bleomycin, radiation, asbestos, silica) to generate a robust fibrogenic response. Parenchymal mutations of key functional regulators (surfactant function, proteostasis, telomere and mitochondrial maintenance) have been abundantly mapped in PF patients (33, 49–52). Mutations of the *SFTPC* gene have been linked to varying degrees of fibrotic disease in adults and pediatric patients (2, 3, 53). In particular, the isoleucine to threonine substitution at position 73 in the SFTPC proprotein represents the most common (25, 26). Previous experimental evidence compellingly showed that mistrafficking of the SP-C^I73T^ mutant leads to epithelial macroautophagy block, polycellular alveolitis, and parenchymal injury consistent of an acute exacerbation (8). Regardless of the model driving fibrogenic response, monocytes/macrophages have been shown to be linked to all phases of the injury process (8, 9, 13, 20, 27). Consistent with this evidence, our RNA sequencing analysis provides comprehensive assessment of the time-related changes in Ly6C^+^ inflammatory monocytes (also pregated as CD11c^-^CD64^-^CD11b^+^) phenotype during initiation and progression of SP-C^I73T^ induced injury. Pathway analysis confirms that peripheral monocytes participate in the initiation of the inflammatory response 3 d post induction (‘cytokine signaling’, ‘innate immunity’), followed by negative enrichment scores for ‘interferon signaling’ and ‘innate immunity’ at 14 d, an observation that supports a time related phenotypic switch in these populations over time. By comparison, signaling pathways related to tissue remodeling (‘collagen formation’, ‘collagen degradation’, and ‘ECM reorganization’) are progressively increased. In support of this notion, gene expression analysis for ‘degradation of ECM’ signaling displays a shift away from signatures canonically seen in fibrosis (*col1a2, col2a1, mmp17* and *mmp24*) (54), while favoring *fn, spp1, timp2, mmp14* and *mmp19* expression. Our data also provides a degree of overlap with other literature describing a pro-remodeling role of monocytes and monocytes-derived alveolar macrophages in fibrogenesis induced by bleomycin (9, 13). By comparison, RNA *in situ* hybridization analysis (*tgfb1)* combined with CCR2 (protein) staining did not fully corroborate these sequencing results. This observation could due to a limitation in our analysis being restricted to evaluation of a small fraction of monocytes (those expressing CCR2). We rather found *tgfb1* expression primarily in the epithelium/mesenchyme, with a number of CCR2^-^ mononuclear myeloid cells expressing *tgfb1*. Consistent with the notion that tfgb1 is involved in fibrosis, we noted significant expression localized within areas of remodeled tissue (55).

While highly informative, our study is not without limitations. For instance, there is clear antibody bias associated with flow cytometric sorting of myeloid populations which could have excluded subgroups of macrophages and monocytes that would not fit the criteria of expression (i.e., Ly6C^int/lo^ expressing cells). To reduce deviation from the literature, we followed previously published protocols for tissue dissociation and phenotypic characterization (9, 35). Furthermore, the methodology utilized for this work (whole body CCR2^KO^ strain) cannot differentiate between “true” resident alveolar macrophages and monocyte populations that acquire such phenotype through maturation. As we move forward, our goal is to provide an unbiased approach that accounts for, or at least attempts to, transcriptional trajectories corresponding to biological processes like monocyte maturation (i.e., single cell sequencing with pseudotime) (56, 57).

A number of reports describe the effects of pharmacological depletion of phagocytic cells (clodronate liposomes) in lung injury and fibrosis (9, 27). Though cleared from the body in a matter of hours (58), we previously presented pathophysiological benefits of intravenous liposome administration lasting up to 14 d post SP-C^I73T^ injury. This was juxtaposed to the protective role of resident alveolar macrophages receiving intratracheal clodronate liposome during the initiation of SP-C^I73T^ induced injury (27). Guided by those results, our RNA sequencing analysis of sorted SigF^-^CD64^-^CD11b^+^Ly6C^+^ monocytes confirms extensive pro-inflammatory and pro-fibrotic activation state. Building on these notions, we opted to focus even further our analysis, by targeting (ablate) a relatively well-established subset of bone marrow-derived inflammatory monocytes (59, 60), identified by their expression of the Monocyte Chemoattractant Protein-1 receptor, CCR2^+^, and examining its effects in SP-C^I73T^ mice. While this monocyte subset accounts for a small proportion of the monocytic milieu, they are known to participate to the initiation of the inflammatory response and thus promote the exacerbation of injury to a fibrotic phenotype (33, 44). The MCP-1/CCR2 axis has been previously shown to be involved in monocyte egression from bone marrow into the peripheral blood and the site of injury (60, 61). Notably, the aberrantly elevated MCP-1 levels found in the BAL of SP-C^I73T^CCR2^KO^ cohorts, indicate that in the absence of adequate monocyte response the pulmonary system compensates by exceeding its normal MCP-1 output. Histochemical analysis of homozygous SP-C^I73T^CCR2^RFP^ mice, in which functional CCR2 was replaced by red fluorescent protein (CCR2^RFP^), indicates that alternative mechanisms of inflammatory monocyte recruitment may be in place. Our findings exclude two established monocyte recruitment pathways (CCL17 and CX_3_CL1), thus leaving non-canonical chemokine axes still in play (CXCL12-CXCR4, CCL20-CCR6 and CCL5-CCR5) (62). Furthermore, immunohistochemical analysis reveals accumulation of mature (CD64), activated (Arg1, iNOS, C1q, p-SMAD2/3) macrophages accumulating from the periphery (CX_3_CR1, CD125/IL5RA). Notably, this aberrant monocyte/macrophage accumulation was solely noted within foci of injury of SP-C^I73T^CCR2^WT^ mice, while their numbers were comparable to those of SP-C^I73T^CCR2^KO^ cohorts in non-inflamed areas. This distinct response is likely connected to ablation of highly destructive monocytes in the initial inflammatory response (3 d post SP-C^I73T^ injury), which dampens subsequent bouts of peripheral immune cell recruitment downstream of the injury (14 d). This notion is supported by our findings of reduced size of remodeled foci and inflammatory cell congestion in the alveolar compartment (in particular eosinophils), improved survival, and lower BAL cell counts in SP-C^I73T^CCR2^KO^ mice. Notable was the surge in Ly6G^+^ neutrophils in SP-C^I73T^CCR2^KO^ mice 14 d post injury, an effects possibly linked to the increase in MCP-1 noted in these cohorts (63), and previously observed in other models of lung injury leveraging CCR2 monocyte depletion (64, 65). Together, these data indicate a clear shift in SP-C^I73T^ induced inflammatory responses following CCR2 ablation, which may be mediated by non-canonical inflammatory paths that will require additional analysis (66).

Examination of injury heterogeneity is vital in the context of PF. To this end, we first confirmed our RNA-sequencing results indicating increases in fibrotic pathways including TGFβ1 *via* histochemical analysis of its downstream effector SMAD2/3 and then studied the spatial correlation of activated (Arg1, iNOS) inflammatory cells with respect to fibrotic foci using RNA *in situ* hybridization. Our data shows reduced numbers of iNOS^+^ and Arg1^+^ cells in the lungs of SP-C^I73^CCR2^KO^ mice, a notion consistent with reduced inflammatory burden in this cohort. While the limited colocalization between activation molecules (Arg1 and iNOS) and *tgfb1* mRNA indicate that neither of those two subsets is involved in direct fibrogenic signaling, we noted increases in the numbers of positive inflammatory cells in areas enriched with *tgfb1*. This observation is indicative of increased recruitment and perhaps communication between parenchymal and inflammatory cells during early fibrotic remodeling, a notion that we aim to test moving forward.

To conclude, we have provided evidence supporting a pro-injury and pro-fibrotic role of peripheral monocytes (both Ly6C^+^ and CCR2^+^) in the initiation and progression of acute inflammatory exacerbations of PF induced by epithelial stress. Using knock out strains and histological techniques we identify activated monocytes/macrophages as disease modifying cells accumulating in proximity to fibrotic foci. In addition, RNA *in situ* hybridization and sequencing analysis demonstrated their potential to promote extracellular matrix remodeling, while highlighting they are not directly involved in fibrogenic factor production (*tgfb1*). Taken together, this work advances our understanding of epithelial-immune cell crosstalk in PF.

## Data Availability Statement

The datasets presented in this study can be found in online repositories. The names of the repository/repositories and accession number(s) can be found here: https://www.ncbi.nlm.nih.gov/geo/, GSE166300.

## Ethics Statement

The animal study was reviewed and approved by IACUC. All mice were housed under pathogen free conditions in AALAC approved facility.

## Author Contributions

AV designed and analyzed all experiments; AV, YT and BA performed all sample collection; SC performed bulk RNA seq analysis; JK, SM, and MFB provided input on study design and interpretation of the results. All authors contributed to the article and approved the submitted version.

## Funding

Research reported in this publication was supported by NIEHS R01ES032553 (AV), VA Merit Review 1I01BX001176 (MB), and NIH R01 HL145408 (MB). JK was supported by NIH K08HL150226, Francis Family Foundation Parker B. Francis Fellowship, and PFF Scholar of the Pulmonary Fibrosis Foundation.

## Conflict of Interest

The authors declare that the research was conducted in the absence of any commercial or financial relationships that could be construed as a potential conflict of interest.

## Abbreviations

AIE, acute inflammatory exacerbations; AT2, alveolar type-2 cell; Sftpc^I73T^ surfactant protein-C I73T mutant; ILD, interstitial lung disease; IPF/PF, idiopathic pulmonary fibrosis; BALF, bronchoalveolar lavage fluid; FACS, fluorescence-activated cell sorting; CCR2, chemokine receptor-2; MoAMs, monocyte-derived alveolar macrophages.
