# Supplementary material for: Role of CCR2+ Myeloid Cells in Inflammation Responses Driven by Expression of a Surfactant Protein-C Mutant in the Alveolar Epithelium
Source: Front Immunol. 2021 Apr 22;12:665818. doi: 10.3389/fimmu.2021.665818 (PMC8101410; doi:10.3389/fimmu.2021.665818)
Supplement: Supplementary file 1 [file DataSheet_1.pdf]

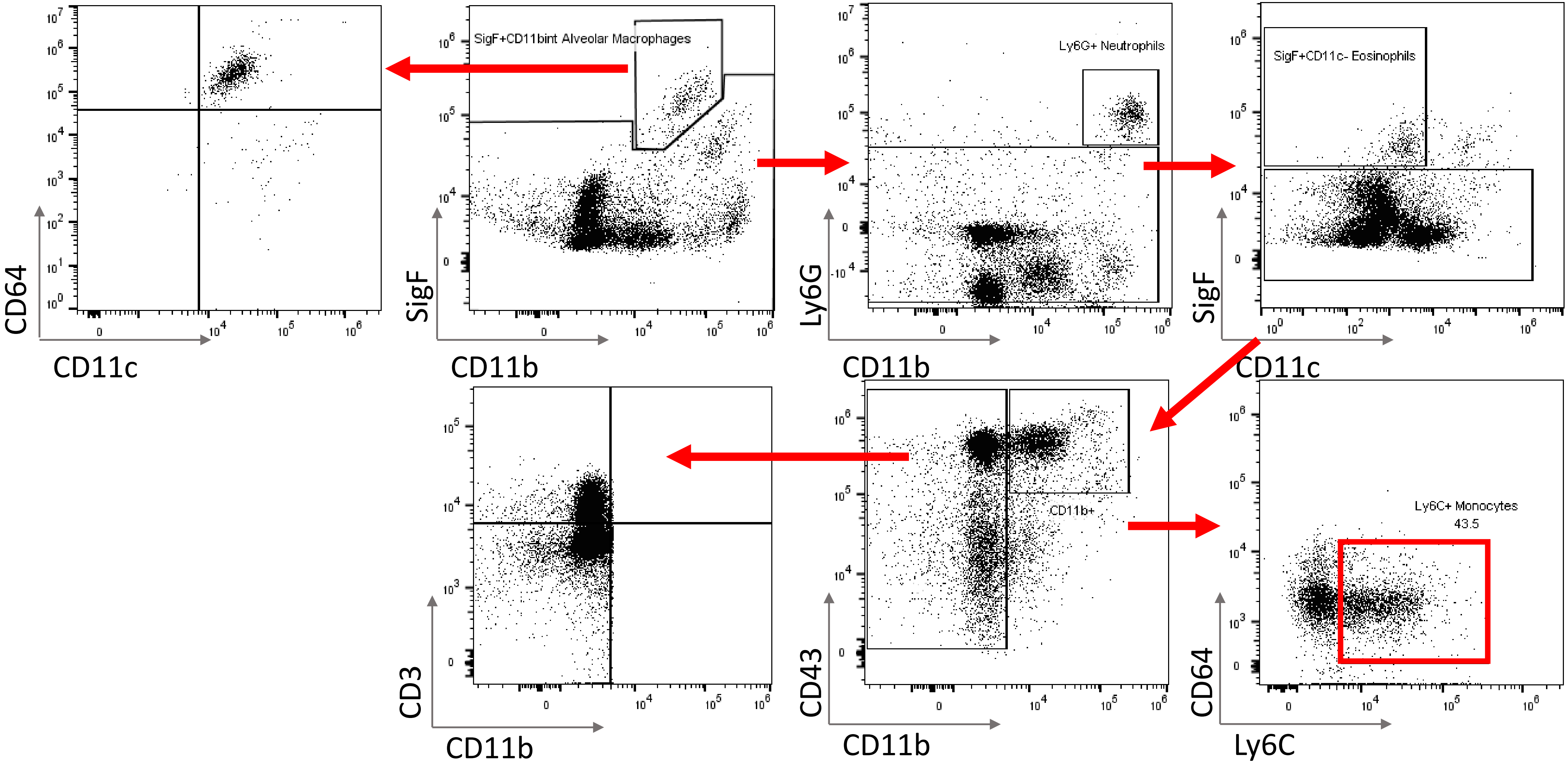

Supplementary Figure 1. Flow cytometric gating strategy. Gating strategy used to identify inflammatory cell subsets in control (CTL, oil treated SP-C^{I73T} mice) or SP-C^{I73T} mice 3 d and 14 d after injury. Cells were gated on CD45^{+} following the exclusion of dead cells and doublets. Populations were identified based on a previously published protocol (Venosa *et al*., 2019). Alveolar macrophages were gated-based SigF^{+}CD11b^{-} expression and confirmed using CD11c, CD64. Sequential gating of neutrophils (CD11b^{+}Ly6G^{+}), eosinophils (SigF^{+}CD11b^{+}CD11c^{-}), lymphocytes (CD3^{+}CD11b^{-}), and monocytes (CD11b^{+}CD64^{-}Ly6C^{+}). Monocytes (highlighted in the red box) were sorted and analyzed via RNA-sequencing.

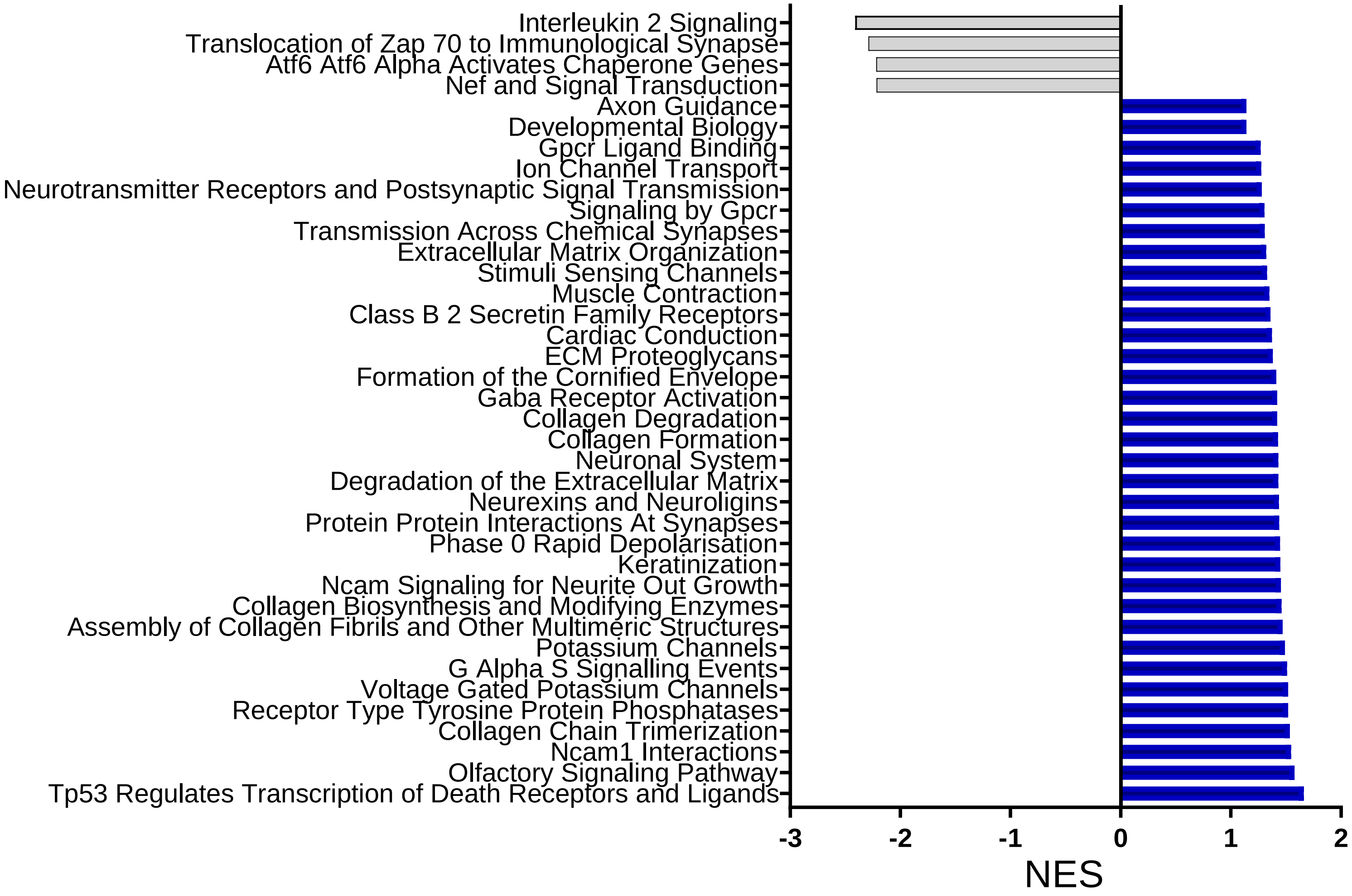

Supplementary Figure 2. Positively and negatively enriched pathways of flow cytometry sorted CD11b$^+$Ly6C^{hi} monocytes following SP-C^{I73T} induced injury. Differentially expressed genes of CD11b$^+$Ly6C^{hi} monocytes isolated 3 d post SP-C^{I73T} induced injury were analyzed using Reactome database to highlight negatively (grey bars) and positively (blue bars) enriched signaling pathways (normalized enrichment score, NES) compared to control (CTL, oil treated SP-CI73T mice). A fold change > 1.5 and false discovery rate [q-value] < 0.05 was considered significant.

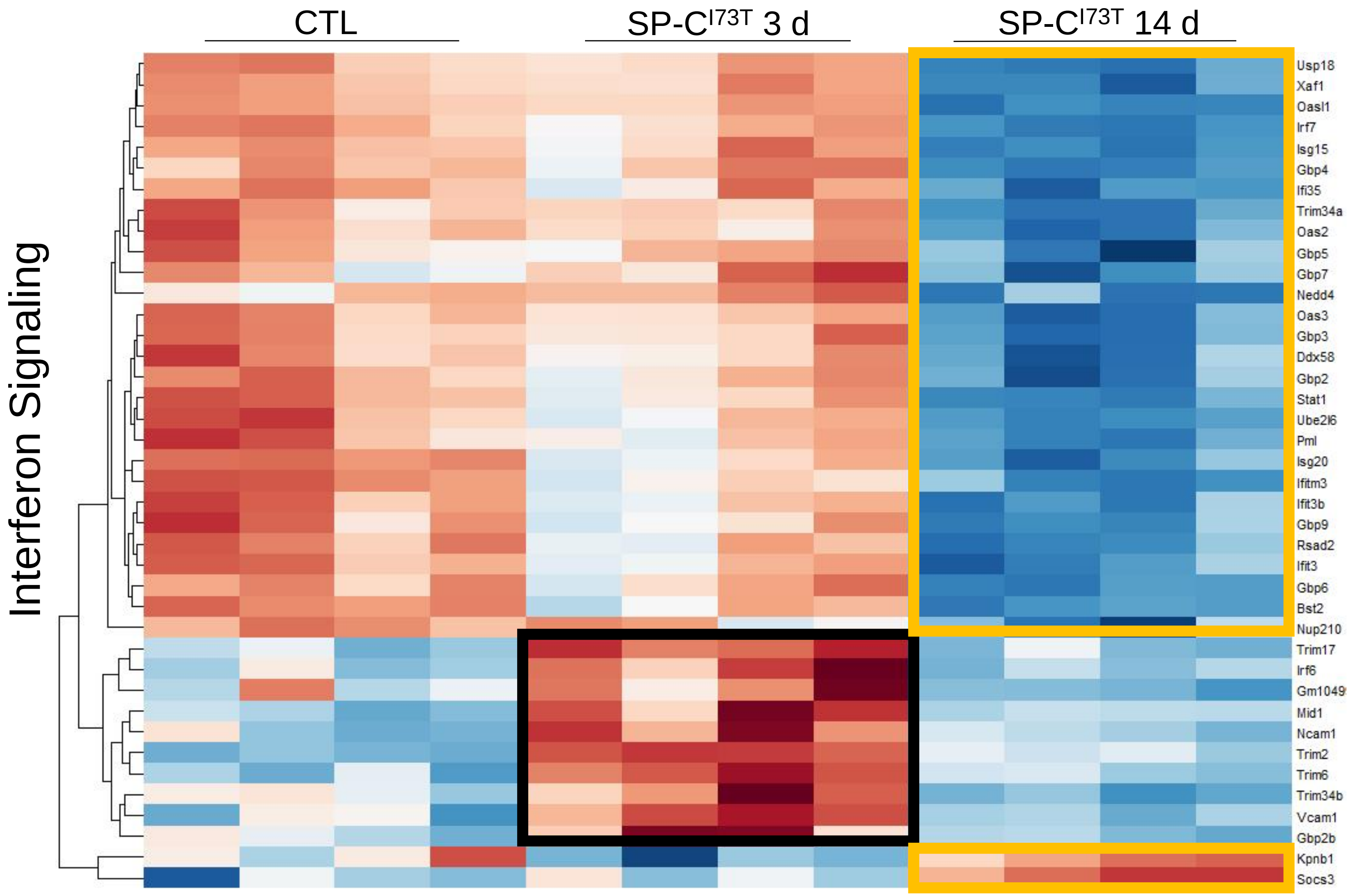

Supplementary Figure 3. Interferon-associated gene expression of flow cytometry sorted CD11b$^+$Ly6C^{hi} monocytes following SP-C^{I73T} induced injury. Differentially expressed genes in CD11b$^+$Ly6C^{hi} monocytes isolated from control (CTL, oil treated SP-C^{I73T} mice) or SP-C^{I73T} mice 3 d and 14 d after injury. Heat-map showing expression of significantly regulated genes involved in 'Interferon Signaling' using Reactome database. A fold change > 1.5 and false discovery rate [q-value] < 0.05 was considered significant. Note **black boxes** represent genes uniquely expressed 3 d after SP-C^{I73T} injury; **orange boxes** represent genes uniquely expressed 14 d after SP-C^{I73T} injury.

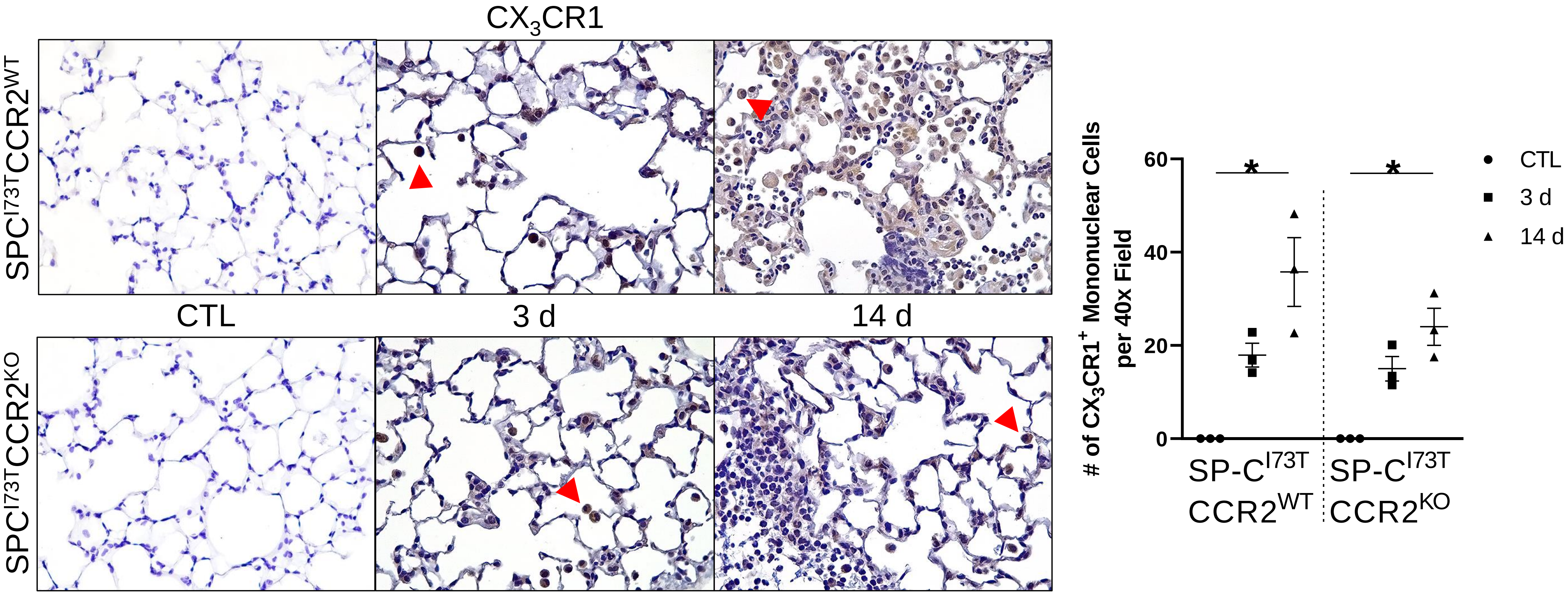

Supplementary Figure 4. Effects of CCR2 monocyte ablation on CX_3CR1 expression following SP-C^{I73T} induced injury. Histochemical analysis of control (CTL, tamoxifen treated SP-C^{WT} or oil treated SP-C^{I73T} mice), SP-C^{I73T}CCR2^{WT} and SP-C^{I73T}CCR2^{KO} lung sections 14 d post injury immunostained with antibody to CX_3CR1. Protein expression was visualized using a DAB Vectastain kit (brown). Arrowheads indicate cells expressing the receptor. Original magnification, 400x. Right panel represents quantification of CX_3CR1 expression in mononuclear cells following SP-C^{I73T} induced injury. 40x images from 5 foci of injury were acquired, acquired and averaged. Data are represented as mean ± SEM (N=3/study group). All analysis was considered significant *$p<0.05$ compared to control mice by One-Way ANOVA, using Tukey post- hoc test.

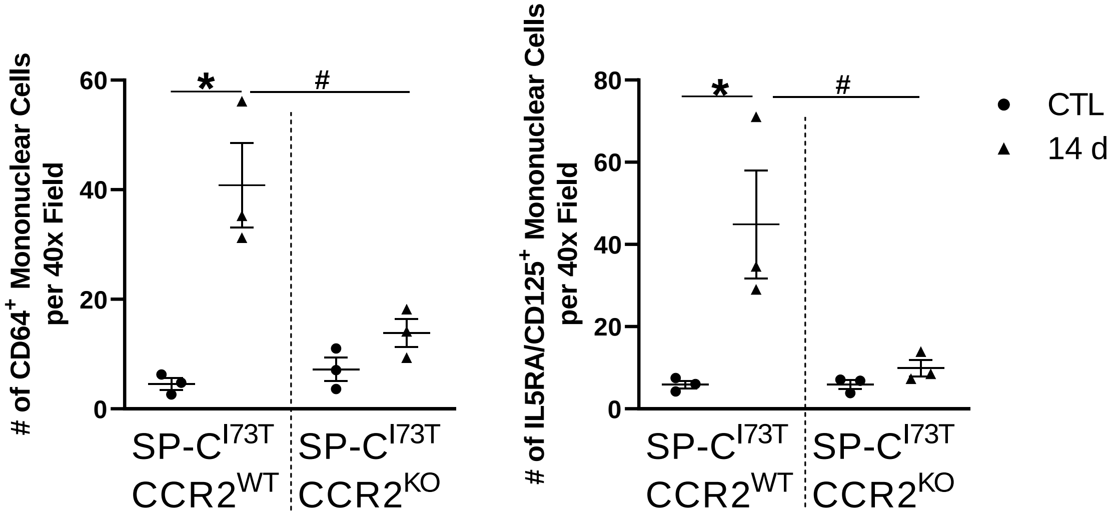

Supplementary Figure 5. Immunohistochemical analysis of monocyte/macrophage maturation and recruitment in SP-C^{I73T} mice. Quantification of histochemical analysis of control (CTL, tamoxifen treated SP-CWT or oil treated SP-CI73T mice), SP-C^{I73T}CCR2WT and SP-C^{I73T}CCR2^{KO} lung sections 14 d post injury immunostained with antibody to CD64 and CD125/IL5RA. Panels depict average number of mononuclear cells present in 40x images acquired from 5 foci of injury. Data are represented as mean ± SEM (N=3/study group). All analysis was considered significant *$p<0.05$ compared to control mice; #$p<0.05$ compared to SP-C^{I73T}CCR2^{WT} mice by One-Way ANOVA, using Tukey post- hoc test.

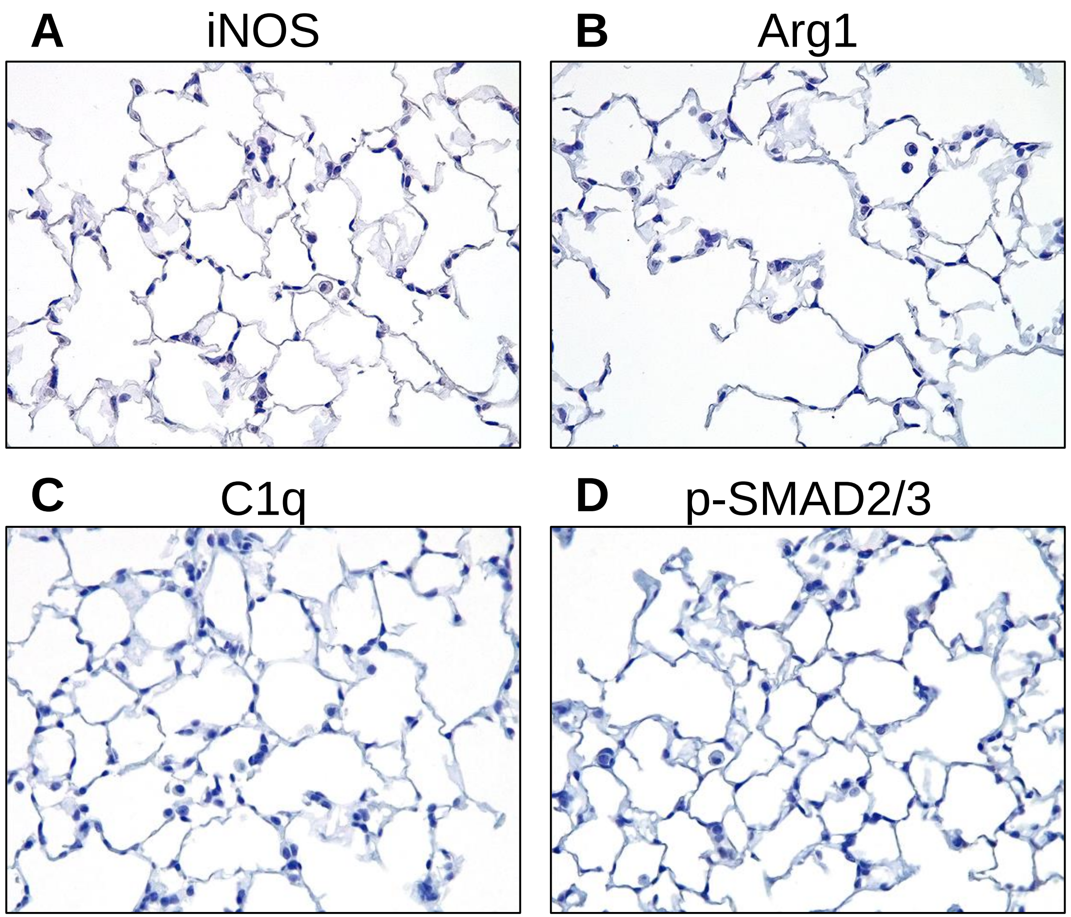

Supplementary Figure 6. Expression of inflammatory and maturity markers in control SP-C^{I73T}CCR2^{KO} mice. Histochemical analysis of oil treated (control) SP-C^{I73T}CCR2^{KO} lung sections immunostained with antibody to (**A**) iNOS) and (**B**) Arg1, (**C**) C1q and (**D**) p-SMAD2/3. Protein expression was visualized using a DAB Vectastain kit (brown). Original magnification, 400x.
